# Structure of glycosylated NPC1 luminal domain C reveals insights into NPC2 and Ebola virus interactions

**DOI:** 10.1002/1873-3468.12089

**Published:** 2016-02-23

**Authors:** Yuguang Zhao, Jingshan Ren, Karl Harlos, David I. Stuart

**Affiliations:** ^1^ Division of Structural BiologyUniversity of OxfordHeadingtonOxfordUK; ^2^ Diamond Light Source LtdDidcotUK

**Keywords:** cholesterol transport, Ebola virus receptor, Ebola virus susceptibility, Niemann–Pick disease type C, NPC1, NPC2

## Abstract

Niemann‐pick type C1 (NPC1) is an endo/lysosomal membrane protein involved in intracellular cholesterol trafficking, and its luminal domain C is an essential endosomal receptor for Ebola and Marburg viruses. We have determined the crystal structure of glycosylated NPC1 luminal domain C and find all seven possible sites are glycosylated. Mapping the disease mutations onto the glycosylated structure reveals a potential binding face for NPC2. Knowledge‐based docking of NPC1 onto Ebola viral glycoprotein and sequence analysis of filovirus susceptible and refractory species reveals four critical residues, H418, Q421, F502 and F504, some or all of which are likely responsible for the species‐specific susceptibility to the virus infection.

## Abbreviations

**DC** domain C

**GP** glycoprotein

**NPC1** Niemann–Pick type C1

**NTD** N‐terminal domain A

**TPC2** two‐pore calcium channel protein 2

Niemann–Pick disease type C is a fatal, neurodegenerative lipid storage disorder resulting from autosomal recessively inherited loss of function mutations in genes NPC1 or NPC2 1. The NPC1 gene encodes an endosomal/lysosomal 13‐pass transmembrane protein with three large luminal domains, namely a cholesterol‐binding N‐terminal domain A (NTD or loop1), domain C (DC or loop2) and domain I (loop3). While NPC2 is a small secreted cholesterol‐binding glycoprotein, it can be translocated to endosomes/lysosomes through its Mannose‐6‐phosphate‐modified glycans 2. Within endosomes/lysosomes, NPC2 traps unesterified cholesterol in a hydrophobic pocket, and hands this over to NPC1 by attaching to luminal domain C and then transferring cholesterol to the N‐terminal cholesterol‐binding domain 3, 4. Loss of function mutations in either NPC1 or NPC2 lead to the accumulation of cholesterol and glycosphingolipids in various tissues and organs, resulting in Niemann–Pick disease neuro‐degeneration as well as lung and liver dysfunction 1.

Apart from its essential role in cholesterol transport, NPC1 has been identified as a critical host entry receptor for filoviruses 5, 6, interacting directly with the viral glycoprotein (GP). Filoviruses, such as Ebola virus and Marburg virus cause haemorrhagic fever with high mortality 7. Filovirus cell attachment is initiated through nonspecific attachment, followed by internalization. When virus containing vesicles are delivered to endosomes, protease cathepsin B/L removes the GP1 cap and mucin domain, exposing the NPC1‐binding sites, and with help of additional factors, such as two‐pore calcium channel protein 2 (TPC2) 8, GP2 drives fusion of the viral and endosome membranes, releasing the viral genetic material into the host cell cytoplasm and initiating viral replication. Only the NPC1 luminal domain C is required for viral glycoprotein binding 5. Intriguingly it has recently been shown that EBOV assembly at the plasma membrane is cholesterol‐dependent and cholesterol might therefore stabilize the virus particle 9.

## Materials and methods

### Protein production and crystallization

Human NPC1 (UniProtKB/Swiss‐Prot 015118) luminal domain C (residues Q387–D618) was PCR amplified from the cDNA (GE Dharmacon, Little Chalfont, UK; clone ID30340517) and cloned into a stable cell line vector pNeoSec 10 in frame with a 3C protease cutting site, monoVenus fluorescent protein and ended with a Rhodopsin 1D4 tag. HEK293S GnTI(−) cells were cotransfected with a pNeoSec‐NPC1‐domain C and a PhiC31 integrase expression vector (pCB92/pgk‐φC31). The polyclonal population resulting from G418 (1 mg·mL^−1^) selection was cultured in roller bottles 11, 12. The conditioned medium containing secreted proteins was passed over Rhodopsin 1D4 antibody‐conjugated Sepharose 4 Fast Flow resin, and eluted by on‐column cutting of the tag using 3C protease. The eluted protein was polished on a Superdex 200 16/60 column, eluted in 10 mm Hepes pH 7.4, 150 mm NaCl buffer and concentrated to 5 mg·mL^−1^. Crystallization screening was carried out using the sitting‐drop vapour diffusion method in 96‐well plates 13 and crystals grown in 30% polyethylene glycol mono‐ethyl Ether 2000 and 0.1 m potassium thiocyanate. Good quality crystals were grown only from protein produced in β1,2‐*N*‐acetylglucosaminyltransferase I deficient (GNTI‐) human embryonic kidney cells, harbouring Man_5_GlcNAc_2_ moieties, whereas Endo F1 de‐glycosylated protein gave poor quality crystals.

### Data collection and structure determination

Crystals were flash frozen in liquid nitrogen, and kept at −173 °C during X‐ray data collection at I04, Diamond Light Source. Data images (exposure time 0.1 s with 30% beam transmission) of 0.1° rotation were recorded on a PILATUS 6M detector (Dectris, Baden‐Dättwil, Switzerland), at a wavelength of 1.7700 Å for the sulfur SAD data set from seven crystals and 1.0675 Å for the native data set from two crystals. Data images were indexed and integrated with Xia2‐3dii 14. The crystals belong to space group *C*222_1_ with two molecules in the crystal asymmetric unit. The structure was determined by sulfur SAD. Sulfur positions, two disulphides and a thiocyanate site, were determined by hkl2map 15. Phasing and initial modelling were done with Phenix‐autosolve 16. Structure refinement and rebuilding used REFMAC 17 and COOT 18. The final model, refined to 2.45 Å resolution, has a R‐factor of 0.218 (R‐free, 0.243) with good stereochemistry. Data collection and structure refinement statistics are given in Table 1.

**Table 1:** Data collection and refinement statistics

Data collection		
Data set	S‐SAD	Native
Wavelength (Å)	1.7700	1.0675
Space group	*C*222_1_	*C*222_1_
Cell dimensions (Å)	*a* = 88.1, *b* = 116.1, *c* = 147.8	*a* = 87.9, *b* = 115.9, *c* = 147.4
Resolution (Å)	73.9–3.10 (3.18–3.10)	70.0–2.45 (2.51–2.45)
Unique reflections	14 134 (1035)	28 062 (2038)
*R*_merge_	0.168 (0.720)	0.063 (–)
CC_50_	1.000 (0.862)	0.999 (0.354)
*<I>*/<σ*I>*	47.7 (4.1)	13.3 (1.0)
Completeness (%)	99.9 (99.4)	100.0 (99.9)
Redundancy	182.7 (16.9)	26.5 (24.4)
Refinement		
Resolution (Å)		70.0–2.45
No. reflections		26 647/1385
*R*_work_/*R*_free_		0.218/0.243
No. atoms		3941
Average *B*‐factor (Å^2^)		51
R.m.s. deviations		
Bond lengths (Å)		0.008
Bond angles (°)		1.5

Numbers in brackets refer to the highest resolution shell of data.

### NPC1–EBOV GP docking

The NPC1DC–EBOV GP (PDB id, 3CSY 19) docking was carried out with Haddock 20 and Gramm‐X 21. Residues 502–504 from NPC1DC, and 86–88, 111–113 141–146 from EBOV GP were provided as the possible interacting residues for restraints. The glycan cap domain of EBOV GP and all the sugar residues of both proteins were removed for docking. The top solutions from both programs predicted a similar binding mode between the two proteins. The putative complex was checked by modelling all the glycosylation sites with Man9GlcNAc2 10 to see if the glycans hinder the formation of the complex and the structure of GP2 dislocated from GP1 after receptor binding modelled to see if it could present its fusion loop to the endo/lysosome membrane. Figures were prepared using PyMOL 22.

## Results and Discussion

### Overall structure of NPC1DC

**Figure 1:**
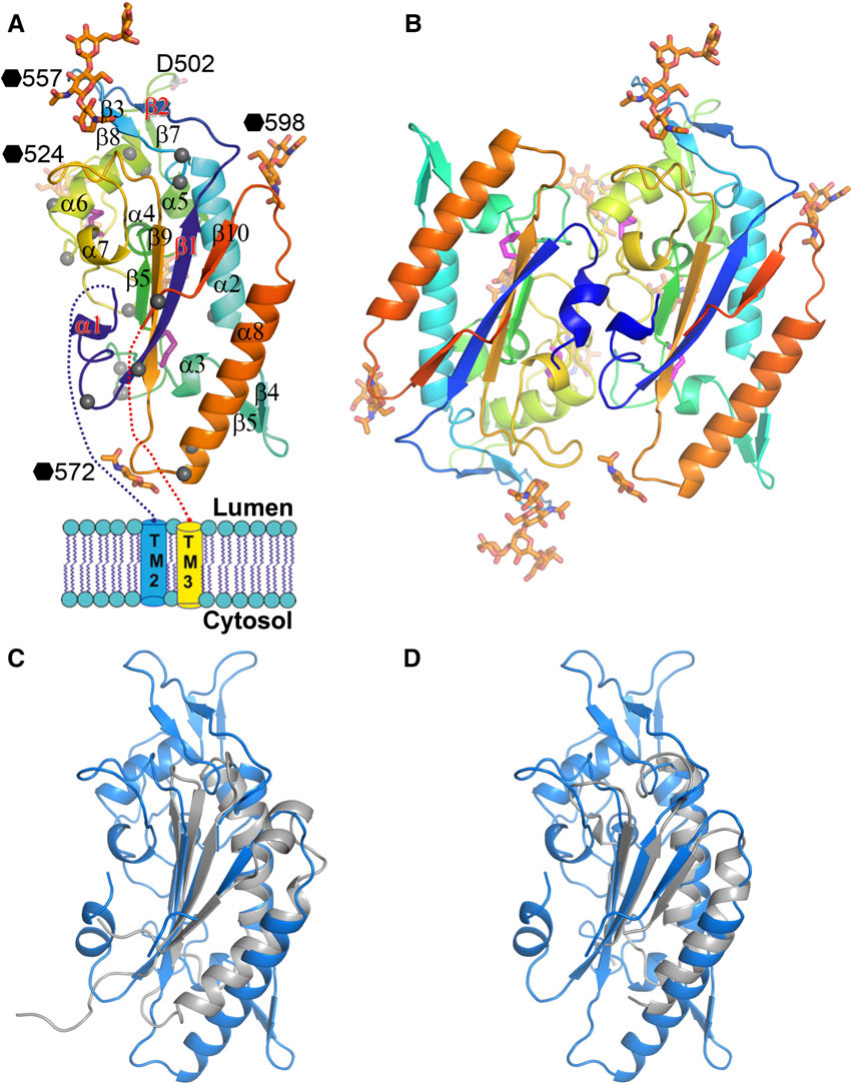
Overall structure of NPC1DC. (A) Cartoon representation of NPC1DC rainbow coloured from N (blue) to C‐termini (red). Grey spheres indicate disease mutation sites. Glycans are shown as orange sticks (labelled with black haxagons), disulphides as purple sticks. (B) The dimer in the crystallographic asymmetric unit. (C) Superimpositions of NPC1DC (blue) with the pore domain of bacterial multidrug efflux transporter MexB (grey) and (D) with the domain 2 of MmpL11 (grey).

To better understand the cholesterol transport mechanism and the Ebola glycoprotein and receptor interaction at atomic level, we determined the structure of human NPC1 luminal domain C (NPC1DC) at 2.45 Å resolution by X‐ray crystallography, utilizing sulfur SAD phasing, refining the model to a reasonable R‐factor with good stereochemistry. Details of the data collection, structure determination and refinement statistics are given in Table 1. The sample produced in HEK293S cells contains residues 387–618, with 15 and 2 residues omitted from the N‐ and C‐terminal transmembrane helices respectively. Residues 392–606 were modelled into the kidney‐shaped molecule that has a core comprising a four‐stranded antiparallel β‐sheet (β1, β6, β9‐ β10) with one side protected by the α2 and α8 helices (Fig. 1A). The convex face of the molecule is outlined by α8, the α8‐β10 loop and the β2–β3 hairpin that connects β1 and α2, while residues linking β5 and β6 and those bridging β6 and β9 form short strands, helices and loose coils stacking in the concave face of the molecule. There are four cysteines (C468, C479, C516 and C533), which are conserved in all species from yeast to human forming two pairs of disulphide bonds. C468–C479 bridges α3 and β6, while C516–C553 anchors the α6–α7 loop to the C‐terminal end of α7. There are seven predicted N‐linked glycosylation sites in the molecule, all of which are glycosylated and five sites have sufficiently clear electron density that at least one glycan residue could be modelled (N478, N524, N557, N572 and N598).

There is one NPC1DC dimer in the crystallographic asymmetric unit (Fig. 1B), however, this is simply an artefact of crystal packing, since gel filtration shows that the molecule is monomeric in solution. A structural similarity search did not reveal any significant hits for the whole NPC1DC, however, the fold of the core structure (four‐stranded β‐sheet, α2 and α8) resembles the pore forming domain of bacterial multidrug efflux transporter MexB (Fig. 1C) 23 and domain 2 of MmpL11 (Fig. 1D) 24 (with rmsds of 2.0 Å for 72 C_α_s and 1.7 Å for 71 C_α_s respectively), both are membrane proteins and have the same orientation with respect to the membrane. The direction of the C‐terminal β10 strand and the role of residue 502 in the interaction with EBOV GP 25 (Fig. 1A) imply that the β4–β5 hairpin and the N‐terminus of α8 are at the membrane proximal end of NPC1DC, a similar orientation to MexB and MmpL11.

### Possible interaction area with NPC2

**Figure 2:**
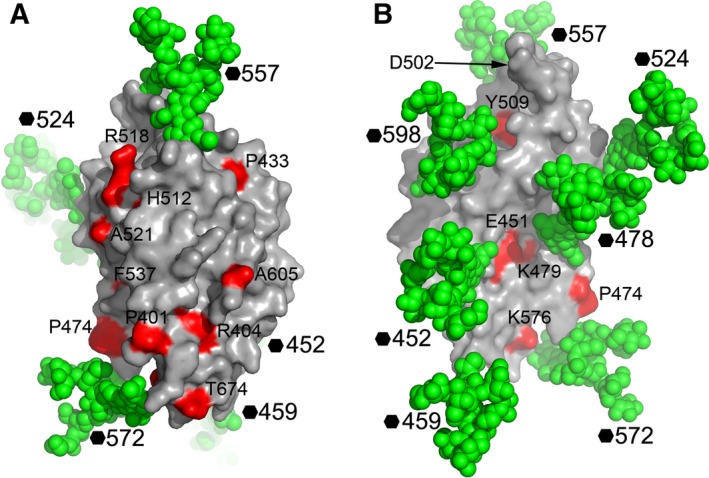
NPC1DC surface mapped with NPC disease mutations. (A) Man9GlcNAc2 is modelled at all seven glycosylation sites of NPC1DC with atoms shown as green spheres on the molecular surface to show glycan‐free areas on the protein. The residues mutated in NPC patients are coloured in red and labelled. The molecule is shown in a similar orientation to Fig. 1A. (B) 180° rotation of (A) showing the back of the molecule. Residue 502, critical for EBOV GP interactions is indicated by an arrow.

In the endo/lysosome, soluble NPC2 binds cholesterol released from endocytosed low‐density lipoprotein and delivers it to NPC1NTD, which transports the cholesterol to the cytosol 4. During the process NPC2 interacts directly with both NPC1NTD and NPC1DC 3; disease‐causing mutations in NPC1DC decrease NPC2 binding 3. We modelled all seven glycosylation sites as Man9GlcNAc2 10 and mapped the disease mutations 26, 27, 28, 29, 30, 31, 32 onto the structure of NPC1DC. As shown in Figs 1A and 2, apart from residues E451, K479, Y509, K576 and T674, these mutations are either buried, and hence likely to cause structural changes, or on a large glycan‐free area of the surface, which is probably involved in interactions with NPC2, K576 and T674 are at the membrane proximal end of the molecule and could alter the orientation of NPC1DC and affect the interaction with NPC2; E451, K479 and Y509, however, are glycan shielded and unlikely to interact with NPC2. Disease‐causing mutations at residues 404 and 518 interfere with the interaction between NPC1 and NPC2 3.

### Interactions between NPC1DC and EBOV GP

**Figure 3:**
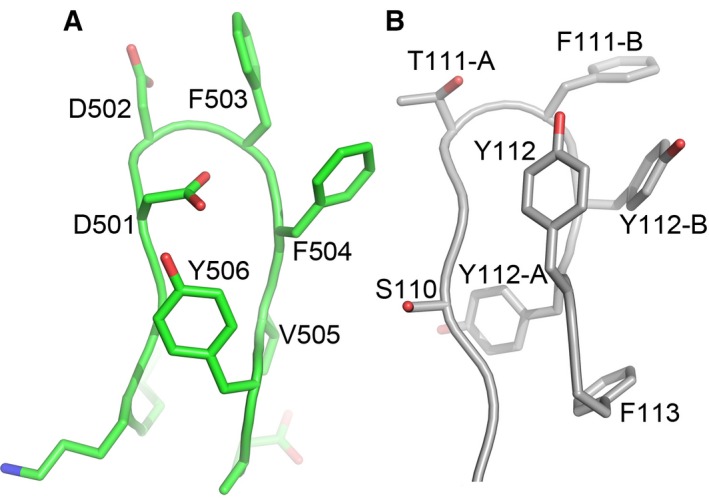
Structural similarity between the β7–β8 hairpin of NPC1DC and the Vh CDR3 of MR78 antibody. (A) β7–β8 hairpin of NPC1DC (500 GDDFFVY 506). (B) Vh CDR3 of the MR78 antibody (110 SGTFYYY 112).

**Figure 4:**
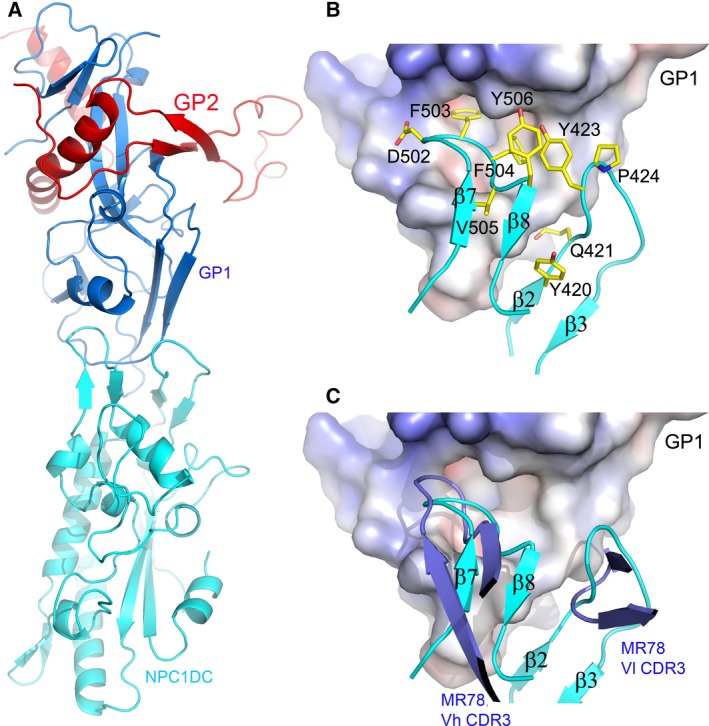
The docked complex of NPC1DC and EBOV GP. (A) Cartoon representation of NPC1DC (cyan) and EBOV GP (GP1, blue; GP2, red) complex. (B) Molecular interface of EBOV GP and NPC1DC. GP is shown as an electrostatic surface, NPC1DC as cyan ribbons with side‐chains as yellow sticks. (C) The docked NPC1DC and EBOV GP complex shows that β7–β8 and β2–β3 hairpins (cyan) are mimicked by interactions of the Vh CDR3 and Vl CDR3 (blue) of the antibody MR78, respectively, with Ebola GP.

**Figure 5:**
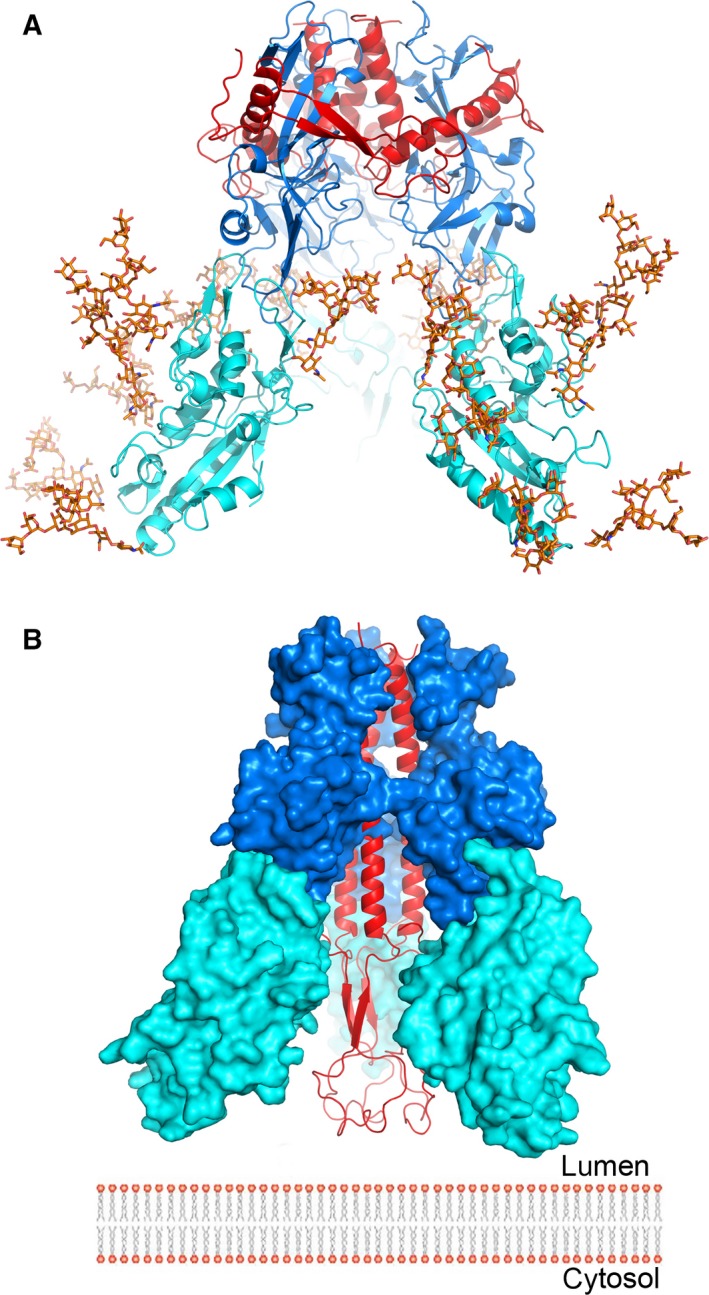
The putative trimeric complex of EBOV GP and NPC1DC. (A) The docked trimetric complex of Ebola GP and NPC1DC. GP1, GP2 and NPC1DC are coloured in blue, red and cyan, respectively. Man9GlcNAc2 is modelled at all glycosylation sites of NPC1DC showing that the glycans do not hinder the formation of the complex. (B) Subsequent to receptor binding, it is proposed that GP2 undergoes conformational changes. Its two helices (residues 553–597) coalesce to present the fusion loop to the endo/lysosomal membrane. The colour scheme is as in (A).

**Figure 6:**
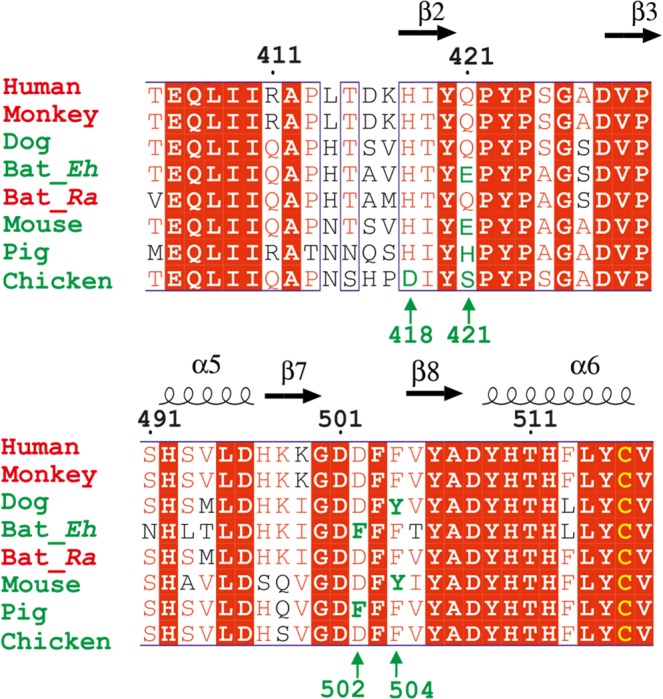
Amino acid sequence alignment of the NPC1DC β2–β3 and β7–β8 hairpin regions. Alignment of NPC1 DC β2–β3 and β7–β8 hairpin regions from Human (*Homo sapiens*), Monkey (*Rhesus macaque*), Dog (*Canis lupus familiaris*), Bat_Eh (*Ediolon helumn*), Bat_Ra (*Rousettus aegyptiacus*), Pig (*Sus scrofa*), Mouse (*Mus musculus*) and Chicken (*Gallus gallus*). Names of susceptible species are coloured in red, and refractory species in green. Numbering corresponds to the full length human NPC1, conserved residues are shown in a red background. Secondary structure elements are labelled on the top. The positions of H418, Q421, D502 and F504 are marked green to indicate the difference in susceptibility to Filovirus infection.

Niemann‐pick type C1 domain C is an essential receptor of all filoviruses 5. It has been reported recently that MR78, an antibody from a human survivor of MARV infection, is cross‐reactive, recognizing the receptor‐binding sites of both MARV and EBOV GPs 33, 34. In addition, African straw‐coloured fruit bats whose NPC1 has a single mutation D502F are nonpermissible to EBOV infection 25. Residue 502 is at the tip of the β7‐β8 hairpin at the presumed membrane‐distal end of NPC1DC (Fig 1A). The NPC1DC β7‐β8 hairpin is remarkably similar to the Vh CDR3 of MR78 in both sequence and structure (Fig. 3). We have modelled the NPC1DC–EBOV GP complex (Fig. 4) by knowledge‐based protein–protein docking. The result is supported by modelling the glycans on the surface of the NPC1DC and the viral GPs structures after receptor binding, which confirms that the binding area is glycan free. Furthermore, the released GP2 would be able to present its fusion loop to the endo/lysosome membrane (Fig. 5). While finalizing this paper, a crystal structure of NPC1DC and EBOV GP complex was published 35, in which the NPC1DC was produced in *E. coli*. The binding mode of the complex appears to confirm our docked complex (see below). In the docked complex the β7–β8 hairpin binds EBOV GP with F503 and F504 nesting in a hydrophobic pocket of EBOV GP similar to F111‐B and Y112‐B of the Vh CRD3 of MR78 (Fig. 4B). Remarkably we find that both the β2–β3 and β7–β8 hairpins interact with EBOV GP by mimicking the Vh CRD3 and Vl CRD3 of MR78 (Fig. 4C). The side‐chain of D502 could hydrogen‐bond to the amino group of F88 of the GP thus a phenylalanine at 502 would cause severe clashes. F504 makes direct contacts with GP V141. A V141A mutation might allow the β7–β8 hairpin to shift, providing enough space to accommodate a phenylalanine at 502, explaining why the V141A mutation can enhance viral entry to F502 bearing cells (Ng *et al*. 25). Interestingly, in chickens all residues in the β7–β8 hairpin are identical to those of primates but they are not susceptible to Ebola virus. Sequence examination reveals chicken‐specific H418D, Q421S mutations in the β2–β3 hairpin (Fig. 6). The side‐chain of Q421 is sandwiched between the β2–β3 hairpin and β7–β9 sheet, possibly hydrogen bonding to either S142 or T144 depending on its conformation on complex formation. In addition, residue 418, located at the edge of a cluster of aromatic residues between the β2–β3 and β7–β8 hairpins, is a histidine in all species except chicken, where it is aspartic acid. This residue makes direct hydrogen bond interactions with Y509 and H510, and the H418D mutation may disturb the conformation of the two hairpins. Thus, mutations at residues 418 and 421 might abrogate EBOV glycoprotein binding.

## Conclusion

The structure of NPC1DC shows the surface area probably involved in the interactions with NPC2 broadening our understanding of cholesterol delivery to NPC1. The species‐specific susceptibility to EBOV infection can be explained by amino acid variations at just four residues, 418, 421, 502 and 504, all of which are located at interface of the putative complex with EBOV glycoprotein.

After submission of this paper, the coordinates of the crystal structure of GP‐NPC1DC complex have been released (5F1B). The overall binding mode between the GP and NPC1 in the docked complex is very similar to the crystal structure. After superimposing the GP structures the Cα positions of the three key receptor residues 502, 503 and 504 differ by 1.3, 1.8 and 2.2 Å respectively.

## Accession codes

Coordinates and structure factors have been deposited in the Protein Data Bank under accession code 5HNS.
